# Network-Structured BST/MBO Composites Made from Core-Shell-Structured Granulates

**DOI:** 10.3390/ma16020710

**Published:** 2023-01-11

**Authors:** Kevin Häuser, Zhiren Zhou, Prannoy Agrawal, Rolf Jakoby, Holger Maune, Joachim R. Binder

**Affiliations:** 1 IAM-ESS, Karlsruhe Institute of Technology, 76344 Eggenstein-Leopoldshafen, Germany; 2 IMP, Technical University of Darmstadt, 64283 Darmstadt, Germany; 3 IIKT, University of Magdeburg, 39106 Magdeburg, Germany

**Keywords:** ceramic composite, FEM, tunability, dielectric behavior

## Abstract

A finite element method (FEM)-based simulation approach to predict the tunability in composite materials was developed and tested with analytical data. These tests showed good prediction capabilities of the simulation for the test data. The simulation model was then used to predict the tunability of a network-structured composite, where the dielectric phase formed clusters in a paraelectric network. This was achieved by simulating a reciprocal core-shell unit cell of said network. The simulation showed a high tunability for this network model, exceeding the tunability of the analytically evaluated layered, columnar, and particulate model. The simulation results were experimentally verified with a Ba_0.6_Sr_0.4_TiO_3_/Mg_3_B_2_O_6_ (BST/MBO) composite, where core-shell granulates were made with a two-step granulation process. These structured samples showed higher tunability and dielectric loss than the unstructured samples made for comparison. Overall, the structured samples showed higher tunability to loss ratios, indicating their potential for use in tunable radio frequency applications, since they may combine high performance with little energy loss.

## 1. Introduction

Dielectric ceramics play an important role in the performance of radio frequency (RF) technology and microwave engineering, due to many device properties depending directly on the used dielectric. While many have been investigated thoroughly, research into new materials and into tailoring their properties is still ongoing [1,2]. Ba_1−x_Sr_x_TiO_3_ (BST) is an important dielectric due to its high permittivity, high tunability, and low dielectric loss when utilized in the paraelectric regime [3,4,5]. To achieve a good compromise between dielectric loss and tunability, composites made from BST and low-loss materials have been under investigation for a long time. One of these low-loss materials is Mg_3_B_2_O_6_ (MBO), which, apart from its very low dielectric loss [6], was also shown to reduce the necessary firing temperature from 1350 °C to 1100 °C [7,8,9,10].

One of the major problems with these composites is the reduction of tunability, caused by the low permittivity and tunability of the MBO. The low permittivity leads to a significant voltage drop over the MBO if no parallel BST pathways are available. Additionally, the non-tunable behavior of the MBO leads to less tuning over the whole composite.

**Figure 1:**
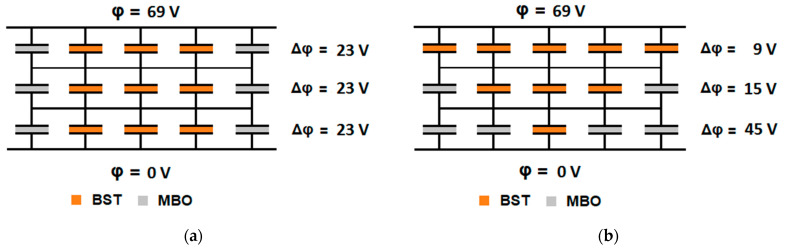
Visualization of the structuring effect on the tunability of the composite. For simplicity’s sake, the voltage distribution in a discrete capacitor network was considered. Furthermore, only one iteration of tuning was applied and the relative permittivity of the MBO was approximated by 0, as ε_r_(BST) >> ε_r_(MBO). The specific tunability of the BST was $1{\;}^{\frac{\%\cdot row}{V}}\;\left(tunability\;per\;electrical\;field\;strength\right)$. The full calculation is shown in the Appendix Calculation A1. (**a**) In a regularly structured composite (corresponds to the pillar model), the MBO has no effect on the tunability. All rows have the same capacitance and the same voltage drop, resulting in a total tunability of 23%. (**b**) In a structured composite with constriction, the BST-poor area (3rd row) is tuned rather strongly. With Kirchhoff’s rules, an overall tunability of about 34% follows.

To reduce this decrease and improve the overall performance of the composite, a network structuring process was investigated. The idea mainly involved creating a regular distribution of MBO clusters in a BST matrix, leading to a fully connected BST network varying in the thickness of its branches. A similar approach has been implemented for porous layers by Giere et al. [11]. This would be advantageous due to the electrical field redistribution into the thinner branches, further increasing the overall tunability. A visualization of this effect is shown in Figure 1.

Investigations and simulations on the dielectric properties of BST/low-loss dielectric composites are not unprecedented. Zhou et al. [12] simulated a two-dimensional structure with randomly distributed BSTs in a low-loss dielectric matrix. Their results showed tunability onsets depending on the BST particle size, ranging from 27% to 45% for particle sizes of 80 nm to 20 nm, respectively. BST particle sizes were not considered in the current study, while no further structural effects were investigated in the study of Zhou et al. Multiple other sources reported similar results for the onset of tunability over BST volume percentage [13,14]. Huber et al. [15] realized and investigated BST/silica core-shell structures, finding a strong decrease in dielectric loss, albeit at a strong loss of tunability.

At the time of writing, the authors of this study are not aware of the existence of other models for the tunability/composition relations of specific 3D-structured BST/low-loss dielectric composites, apart from the shown MEMA model from Sherman et al. [16].

## 2. Materials and Methods

### 2.1. Simulation

To obtain an estimate of magnitude for the presented structuring effect for a core-shell-structured composite, the tunability was modeled with a finite element simulation. The model was implemented in ABAQUS based on the heat transport model. This was implemented due to the similarities between the corresponding differential equations:

$$\vec{q}=-k\nabla T$$

for the heat conductivity depending on the gradient of the temperature

$$\vec{p}=-\varepsilon \nabla \varphi =\varepsilon \vec{E}$$

for the polarization depending on the gradient of the electrical potential

An important adaption that had to be made was the dependence of permittivity and therefore tunability on the local potential gradient.

$$\varepsilon =\varepsilon \left(\nabla \varphi \right)$$

the permittivity depending on the local potential gradient.

This adaption was implemented with a material-specific user subroutine (UMAT), which is shown in the Appendix A Code A2.

The resulting model allowed for the prediction of the permittivity of a structured composite made from two defined materials, each possessing a standard permittivity and a reduced permittivity for a defined difference of boundary conditions, which corresponded to an external tuning voltage.

The values used for the pure materials were 1300 and 7 for the relative permittivity of the BST and MBO, respectively, and 0% and 40% for the tunability of the BST and MBO at full tuning voltage.

The model can then make predictions for the structured composites of these two materials with a differing of boundary conditions, corresponding to the presence or absence of an external tuning voltage. From these values, the tunability can be calculated for a chosen structure.

To validate this model, the column and layer model by Sherman et al. [16] were analyzed. These models predict the permittivity of a composite for different structures and compositions. They do not predict the behavior under the tuning voltage. The best approximation that could be made here was the assumption that the BST would be fully tuned. This allowed us to predict the overall tunability by adjusting the permittivity of the BST according to full tuning. Therefore, for cases of strong voltage redistribution out of the BST, this approximation would lead to large errors.

### 2.2. Experimental

In order to realize the simulated core-shell structure, a two-step granulation structuring process was developed. The materials were prepared as described in a previous study, which also showed the characterization results of the materials [17]. In the first step, an MBO granulate was prepared as the core structure of the composite. For this, the prepared powders were separately milled with isopropyl as the dispersion agent in an attrition mill, with 200 µm of Y-stabilized zirconia grinding the media down to a d_50_ of 100 nm. Afterwards, the milled MBO powder was dispersed in isopropyl alcohol until a suspension with 17.0 wt% solid percentage was obtained. This suspension was spray-dried with an inlet temperature of 180 °C and a spray rate of 10 mL/min to form homogeneous granulates.

The BST powder was subsequently mixed with the obtained MBO granulates and dispersed in H_2_O at 17.0 wt%. This mixture was then spray-dried with an inlet temperature of 250 °C and a spray rate of 10 mL/min to obtain BST-coated MBO granulates.

Since other composite effects other than the structuring might influence the final performance of the manufactured samples, they were not directly compared to the simulation, but instead, to a set of comparison samples. These were prepared with the same starting materials, but instead of the two-step granulation process, they were made by mixing in a planetary ball mill at 200 rpm for 2 h, with isopropyl as the dispersion agent and 3 mm of Y-stabilized zirconia milling balls. Afterwards, the powder was made into granulates by spray-drying with an inlet temperature of 180 °C and a spray rate of 10 mL/min, with a 17 wt% solid percentage.

In order to measure the dielectric properties, pellets were made from the coated granulate with uniaxial pressing at 150 MPa. They were sintered afterwards at 1050 °C for 2 h, with a heating rate of 5 K/min and a cooling rate of 10 K/min, to ensure sufficient density (>90% of the theoretical density) for voltage resistance and interconnectivity of the particles.

The microstructure was investigated with a scanning electron microscope (Supra 55, Zeiss, Oberkochen, Germany).

The images were taken at 10 kV of acceleration voltage with an AsB detector in order to distinguish the different phases in the composite. Image analysis was conducted via ImageJ to find the MBO volume percentages and BST grain sizes. A threshold was applied to distinguish BST and MBO, which gave an estimate of the dielectric content. Adjusted Voronoi splitting was applied to compensate for closely packed grains, and the resulting image was analyzed with the ImageJ cell analysis plugin to obtain the grain size distribution.

Dielectric properties were investigated by means of impedance analysis with a Keysight E4991B (Keysight, Santa Rosa, CA, USA). The measurements were conducted at 13.56 MHz, with an AC amplitude of 1 V and a DC bias voltage of up to 1.1 kV. The DC bias was applied with a Keithley 2410 1100 V sourcemeter, which was separated from the radio frequency setup with a pspl5531 bias tee.

## 3. Results and Discussion

### 3.1. Simulation

**Figure 2:**
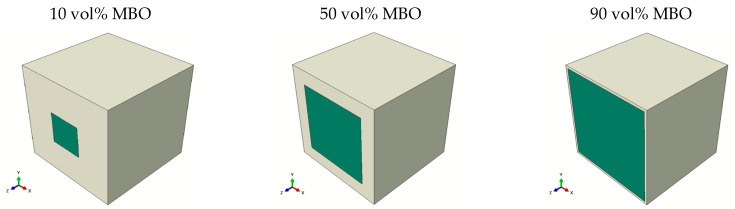
Visualization of the simulated columnar structure for different MBO percentages, which are depicted in green, while the BST ceramic is shown in white. The boundary conditions, which correspond to the applied voltage, were applied along the z-axis (from front left to back right).

The *columnar model* was the simplest of the investigated structures. It was represented by a parallel connection of two capacitors, each corresponding to one material. This resulted in the full difference in boundary conditions applying to both materials, leading to the full tuning of the paraelectric phase. The structure is represented in Figure 2. The boundary conditions were applied to the front left and the bottom right.

**Figure 3:**
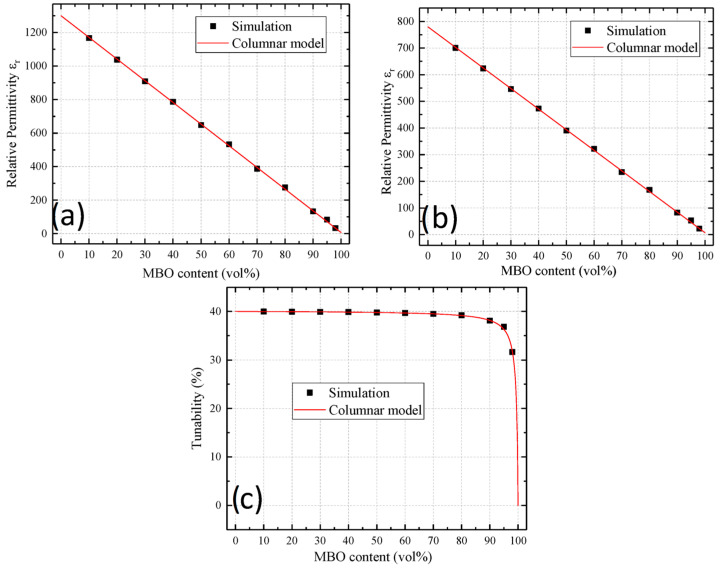
Comparison of the non-tuned permittivity (**a**), the tuned permittivity (**b**), and the resulting tunability (**c**) of the presented simulation (black points) and the columnar model of Sherman et al. [16] (red line). Since no voltage redistribution took place in the columnar model, the model of Sherman was assumed to predict the tunability correctly for this structure. No significant deviation between the model and the simulation was observed.

The results of the simulation for the columnar model are shown in Figure 3. The graphs in the upper row show the permittivity of the composite in the untuned and tuned states, respectively. The bottom graph shows the resulting tunability. The simulation data (black dots) were compared with the columnar model of Sherman et al. [16] (red line). Since full tuning of the BST was assumed in the columnar model, a linear decrease in permittivity over the MBO content was observed, reduced by the tunability factor for the tuned sample. The simulation illustrated the constant tunability up to a high MBO content, where the properties of the MBO started to dominate the overall behavior.

**Figure 4:**
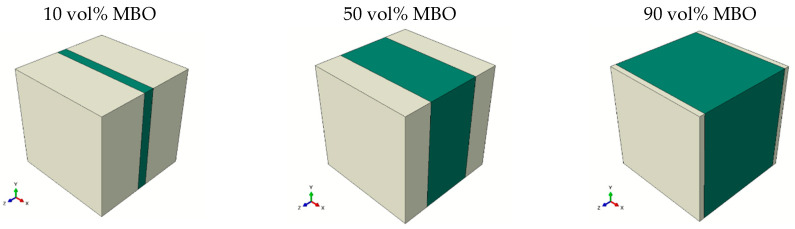
Visualization of the simulated layered structure for different MBO percentages, which are depicted in green, while the BST ceramic is shown in white. The boundary conditions, which correspond to the applied voltage, were applied along the z-axis (from front left to back right).

*The layered model* was a rather simple structure as well. It was represented by a serial connection of two capacitors, each corresponding to one material, as depicted in Figure 4. This resulted in a strong voltage drop over the MBO, significantly reducing overall tunability. It can, therefore, be used to investigate the ability of the simulation to adjust for voltage redistribution.

**Figure 5:**
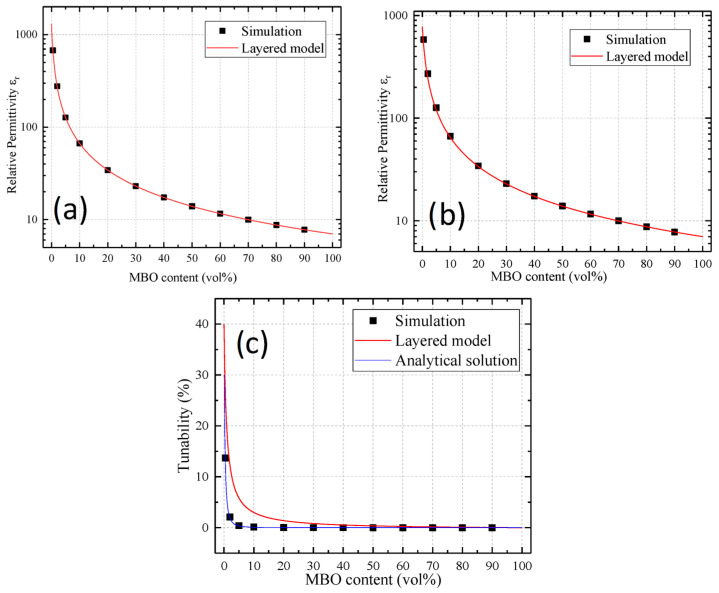
Comparison of the non-tuned permittivity (**a**), the tuned permittivity (**b**), and the resulting tunability (**c**) of the presented simulation (black points) and the layered model of Sherman et al. [16] (red line). Since the layered model features maximal voltage redistribution, the model of Sherman is assumed to strongly overestimate the tunability of the composite. As the layered model is comparatively simple and can be treated as a simple serial connection of two capacitances, the voltage redistribution can analytically be accounted for. This corrected prediction is shown as the blue line in the tunability graph. As can be seen, the values fit well to the simulated data.

The results for the layered model are shown in Figure 5. The graphs in the upper row show the permittivity of the composite in the untuned and tuned states, respectively. The bottom graph shows the resulting tunability. The simulation data (black dots) were compared with the layered model of Sherman et al. [16] (red line). To see the general behavior of the permittivity for the layered model, both permittivity graphs show the permittivity in logarithmic scaling. While no obvious deviation can be made out, the tunability graph shows significant differences for the simulation and the layered model. This was to be expected, since the layered model did not take voltage redistribution into account, but instead, assumed full tuning over the BST. In reality, most of the voltage will drop over the MBO, even for low MBO percentages. To obtain a useful comparison to the simulated data, an analytical solution was calculated (explained in Appendix A Calculation A3). When comparing the simulation to this solution, a good fit could be made out, indicating a good sensibility of the simulation in regard to voltage redistribution.

**Figure 6:**
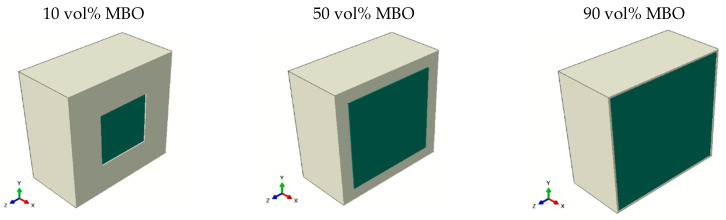
Visualization of the simulated core-shell structure for different MBO percentages, which are depicted in green, while the BST ceramic is shown in white. Symmetrical cross-sections are shown so that the MBO can be seen. The complete object was formed by mirroring across the shown interface (front right). The boundary conditions, which correspond to the applied voltage, were applied along the z-axis (from front left to back right).

The *core-shell model* was a comparatively complex model, including the voltage redistribution within the x–y layers. It featured a change of channel width for the BST. To reduce the required simulation resources, a reciprocal structural object was chosen, which is depicted in Figure 6. The reason for using a cube instead of a sphere for the MBO inclusion was mostly to be able to simulate high MBO percentages without complex material redistribution within the simulated object. The boundary conditions were applied to the front left and the bottom right.

**Figure 7:**
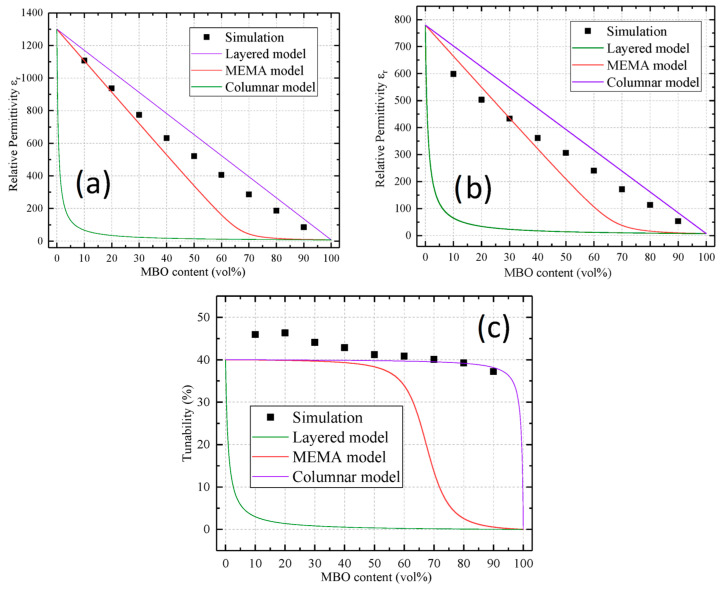
Comparison of the non-tuned permittivity (**a**), the tuned permittivity (**b**), and the resulting tunability (**c**) of the presented simulation (black points), both of the presented models of Sherman et al. [10] (green and purple lines), and the MEMA model (red line).

The results of the simulation for the core-shell model are shown in Figure 7. The graphs in the upper row show the permittivity of the composite in the untuned and tuned states, respectively. The bottom graph shows the resulting tunability. The simulation data (black dots) were compared with a particle model of Sherman et al. [16] (red line), which assumed the homogenous distribution of particles (called MEMA model), the layered model (green line), and the columnar model (purple line). None of these attempted to describe a core-shell structure, which is why strong deviations are to be expected. Instead, they are shown here to compare the simulated structure to the expected behaviors of different kinds of structuring. The permittivities of the core-shell structures were close to the particulate model for low MBO contents and approached the columnar model for high MBO contents. This was expected, due to the layout of the structure (compare Figure 6). The tunability did show a higher value than that of the pure BST for low to medium MBO percentages, which can be attributed to the structuring effect explained in Figure 2. Even for high MBO percentages, the tunability did not drop significantly, especially when compared to the particulate model. This was due to the retention of BST percolation, which would be lost in a particulate composite above the percolation threshold.

The simulated retention of high tunability with high MBO contents is highly desirable for application in tunable dielectric devices. In order to realize the required high percolation threshold, a transition from particulate to structured composites could prove fruitful, in which the dielectric phase is clustered and surrounded by thin BST paths. In the following chapter, this idea will be realized experimentally and verified.

### 3.2. Experimental

**Figure 8:**
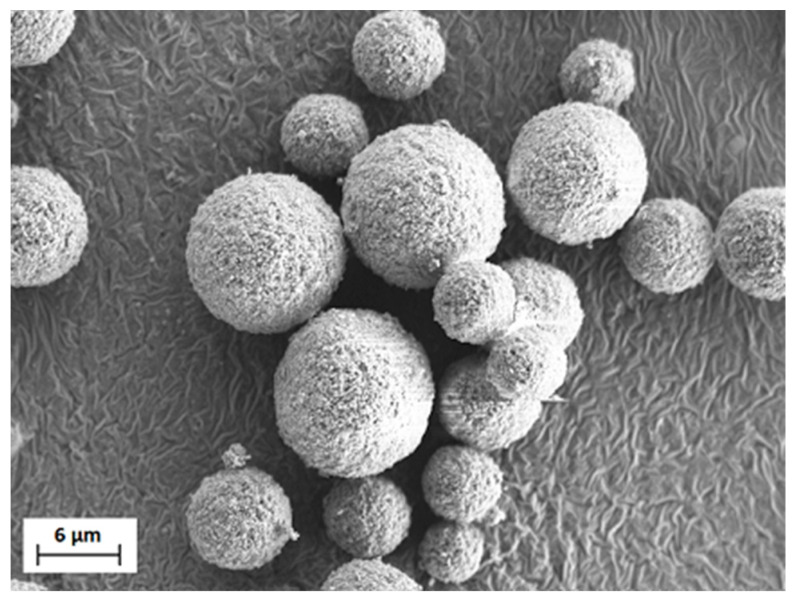
SEM image of the pure MBO granulates.

**Figure 9:**
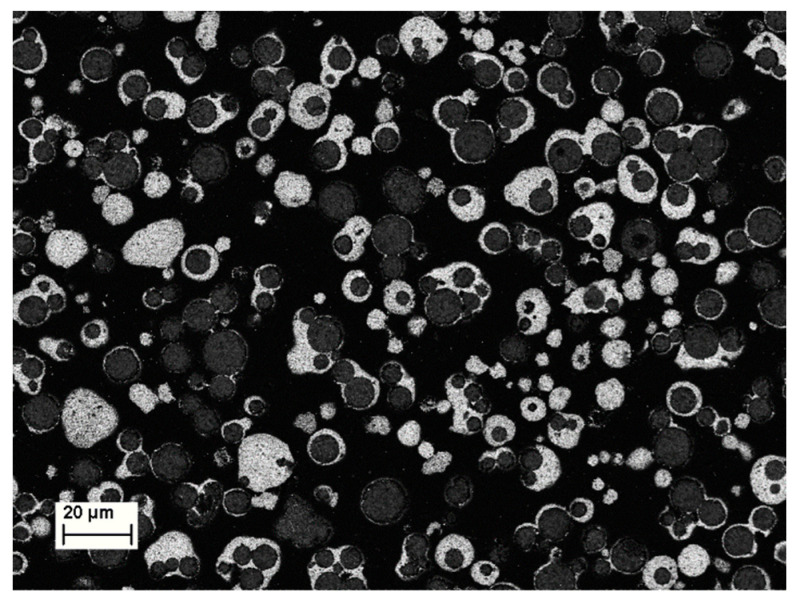
SEM image of a milled cross-section of the coated granulate. MBO is shown in dark grey, BST is shown in white, and the background resin is shown in black.

The pure MBO granulates were investigated by SEM to ensure reasonable homogeneity and the absence of big agglomerates. An exemplary image is shown in Figure 8. After the coating step, some of the composite powder was embedded in resin and milled to obtain a cross-section of the coated granulates. An image of such a cross-section is shown in Figure 9. 

The samples with nominal compositions of 30, 50, and 70 vol% MBO were analyzed with scanning electron microscopy and image analysis to investigate their actual compositions and the grain sizes of the BST. The heights of the prepared samples were measured as well, in order to correct the dielectric measurements for the difference in electric field strength for a set applied voltage. The data of the structured samples are shown in Table 1, and the data of the unstructured comparison samples are shown in Table 2. At a low MBO content, the BST grains of the structured samples were larger, while for higher MBO contents, the BST grains were of comparable size.

**Figure 10:**
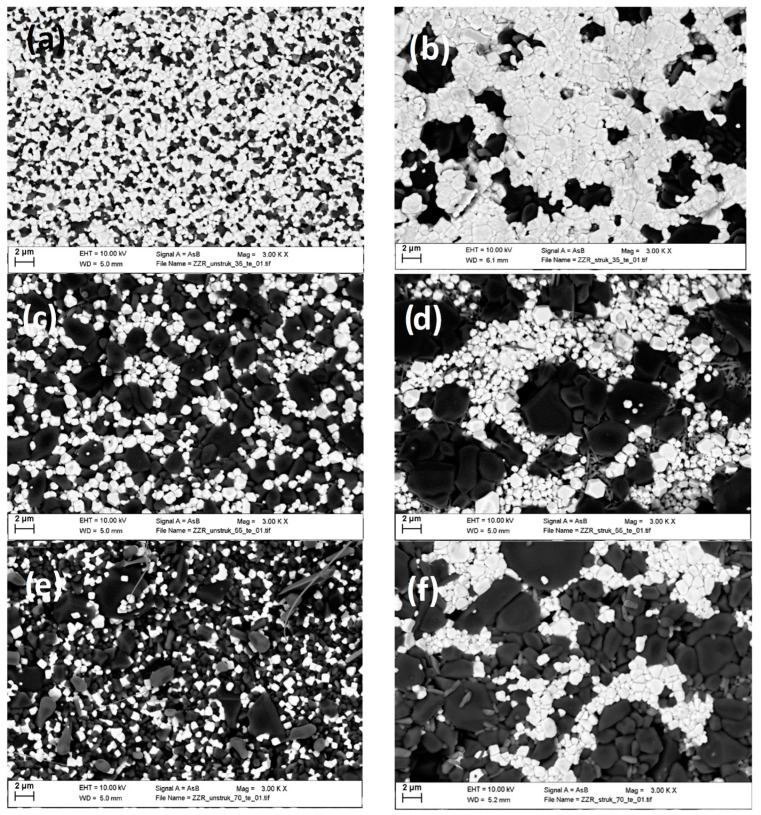
Close-up SEM images of the microstructure of the prepared samples, with BST being shown in white and MBO in grey. Images (**a**), (**c**), and (**e**) show the unstructured samples with MBO contents of 40 vol%, 48 vol%, and 64 vol%, respectively. Images (**b**), (**d**), and (**f**) show the structured samples with MBO contents of 35 vol%, 55 vol%, and 73 vol%, respectively. These images hint at a stronger clustering of the MBO in the structured samples, caused by the core-shell structuring of the granulates.

**Figure A1:**
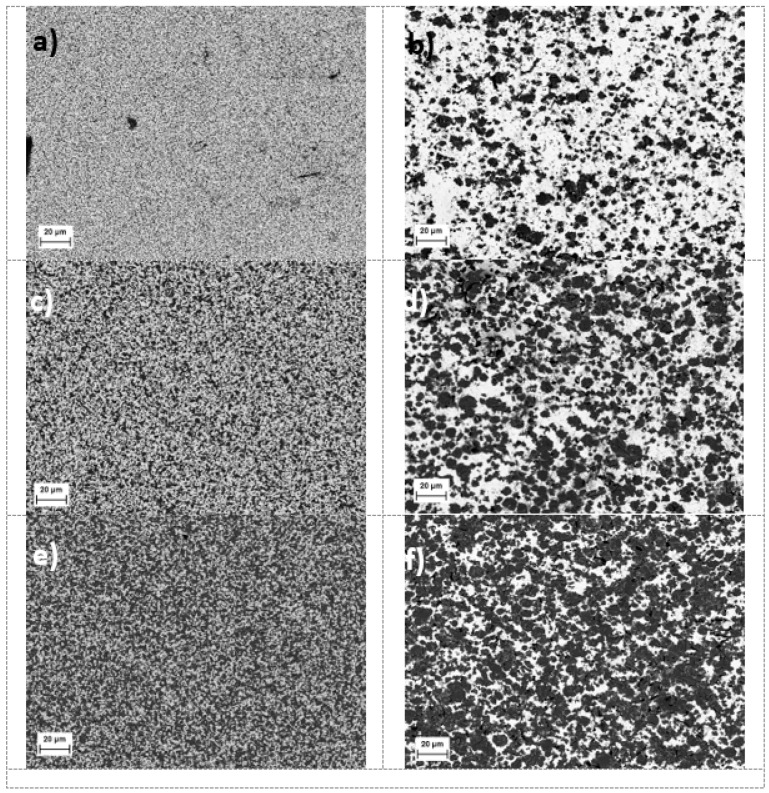
SEM images of the microstructures of the prepared samples, with BST being shown in white and MBO in grey. Images (**a**), (**c**), and (**e**) show the unstructured samples with MBO contents of 40 vol%, 48 vol%, and 64 vol%, respectively. Images (**b**), (**d**), and (**f**) show the structured samples with MBO contents of 35 vol%, 55 vol%, and 73 vol%, respectively. These images hint at a stronger clustering of the MBO in the structured samples, caused by the core-shell structuring of the granulates.

Figure 10 shows exemplary SEM images of the pressed and sintered pellets for the structured and unstructured samples for different MBO contents. Apart from the change in composition, a strong clustering of MBO in the structured samples can be observed. While a purely 2D visual inspection cannot truly evaluate the establishment of a percolating BST network, this clustering indicates a successful transition of the core-shell-structured granulates into an according microstructure of sintered compacts. The images are shown again, with lower magnification, in the Appendix A in Figure A1.

**Figure 11:**
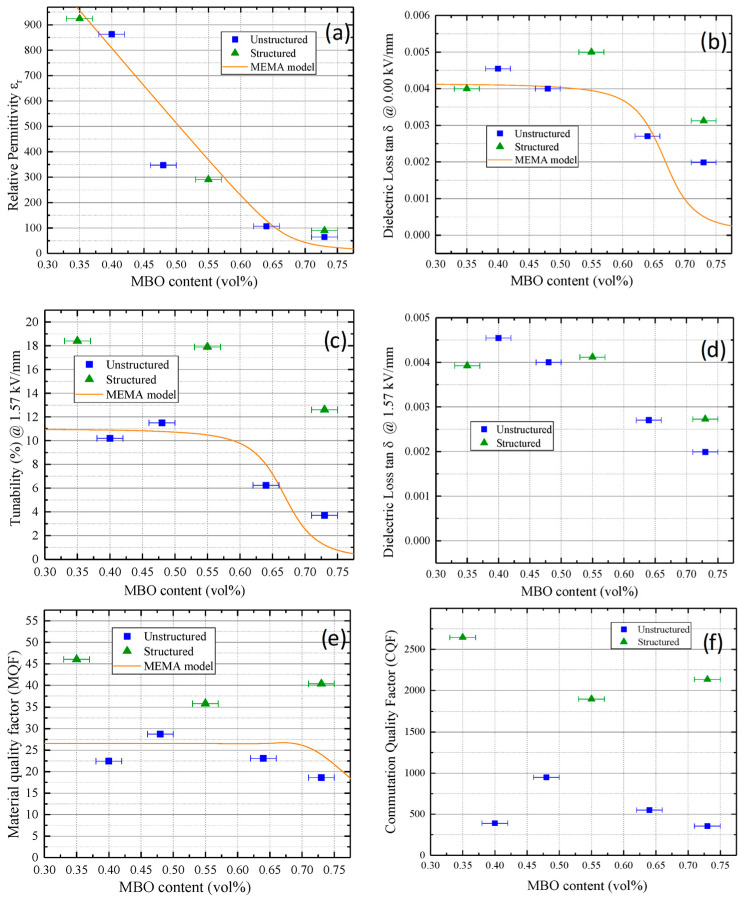
Relative permittivity (**a**), dielectric loss without tuning voltage (**b**), tunability (**c**), dielectric loss with a 1.57 kV/mm tuning voltage (**d**), material quality factor (MQF) (**e**), and commutation quality factor (CQF) (**f**) of the structured and unstructured composites at 13.56 MHz and 48 °C. Where available, the MEMA model for particulate composites by Sherman et al. [16] was fitted for comparison (orange line).

The results of the dielectric measurements are shown in Figure 11, comparing the results of the structured samples with the results of the unstructured samples. The measured data was corrected for the different thicknesses of the pellets, and therefore, dielectric field strengths, by selecting the maximal measured field strength of the thickest sample and comparing all measurements at this field strength. Subfigure (a) shows the permittivity in the untuned state. While the unstructured samples lightly show more exponential behavior, no major difference was observed between the structured and unstructured samples. Subfigure (b) shows the dielectric loss in the untuned state. For low MBO contents, the losses were comparable, but from roughly 50 vol% MBO on, the unstructured samples showed lower dielectric losses. In subfigure (c), the tunability is shown for a tuning voltage of 1.57 kV/mm. Here, significantly higher values for the structured samples were observed, particularly for high MBO contents, where the unstructured samples showed almost no remaining tunability. Subfigure (d) shows the dielectric loss again, but this time at a tuning voltage of 1.57 kV/mm. While the dielectric loss of the structured samples still exceeded that of the unstructured ones, the difference was smaller than in the measurement without any tuning voltage. To get an impression of the overall performance of both batches, their material quality factor and commutation quality factor were compared, as shown in subfigures (e) and (f), respectively. The material quality factor indicates the performance under small tuning voltages, while the commutation quality factor also factors in the performance under high tuning. Their formulas are given in the Appendix A Calculation A5. The structured samples outperformed the unstructured samples for all MBO contents in the material quality factor due to their higher tunability. This difference even increased for the commutation quality factor, where the reduced dielectric losses in the tuned state additionally contributed to the performance of the structured samples.

Overall, the measured tunabilities reached as high as 50% for the 35 vol% MBO structured sample at an electric field strength of 2.2 kV/mm. At the same time, the sample only featured a dielectric loss of 0.013, resulting in favorable properties for usage in tunable dielectric ceramics. A tabular overview of the measured properties can be found in the Appendix A in Table A1. A direct comparison of the simulation results and the experimental results was omitted due to the somewhat arbitrary input values of the simulation, namely the values of the pure materials, which did not line up in the interpolation with the composite materials (see [17]).

Comparing these results to literature, most sources focused on the influence of structuring on dielectric properties in an electrically static case. Buscaglia et al. [18] fabricated core-shell-structured dielectric ceramics and observed the modulation of the relative dielectric permittivity by the sizes of the core particles. They observed significantly higher permittivities and dielectric losses, which could be explained by their doubly ferroelectric composites in contrast to the paraelectric/dielectric composite investigated in this work. For those measuring tunability, Jylhä et al. [13] investigated polymer/BST composites, finding significantly lower tunabilities and dielectric losses than shown in this work for comparable permittivities. They used much lower electric field strengths, which makes a comparison with this work difficult.

Huber et al. [15] encased their paraelectric phase in silica but did not fully densify it, causing significantly lower permittivies, higher dielectric loss, and much lower tunabilities, albeit once again at lower electric field strengths.

Comparable electric field strengths were used by Su et al. [3]. In their work, the Mg-doped BST showed a higher dielectric loss and permittivity with lower dielectric loss. Their samples were measured at 10 kHz, compared to the 13.56 MHz in this work, and they added, at most, 5% Mg, making a comparison over the whole composition range impossible.

## 4. Conclusions

A FEM based simulation model to predict the tunability of a two-phase composite material over composition was developed and tested for two known structures. Afterwards, it was used to investigate the behavior of a reciprocal core-shell cell with the dielectric material as the core. These simulations showed higher tunabilities for any dielectric content for this core-shell structure than for the two test structures or for the analytically predicted behavior of a particulate composite. The predicted tunability of the core-shell structure even exceeded the tunability of the pure paraelectric phase for low dielectric contents. To verify these simulations experimentally, composites were made from Ba_0.6_Sr_0.4_TiO_3_ as the paraelectric phase and Mg_3_B_2_O_6_ as the dielectric phase. The core-shell structures were prepared using a two-step granulation process, where MBO granulates were coated with BST in the second step. This powder was made into ceramic pellets and compared with unstructured samples, which were made from stochastically mixed MBO and BST powders. The structured samples showed slightly higher dielectric loss and significantly higher tunability, resulting in an overall strong increase in material performance, as shown by the material quality factor and the commutation quality factor. These results indicate a potential for network-structured ceramic composites in radio frequency applications, building upon the already existing advantages of ceramic composites, such as lower sintering temperatures and lower dielectric losses, when compared to pure paraelectric materials.

## 5. Patents

K. Häuser, P. Agrawal, R. Jakoby, H. Maune, J.R. Binder. Kompositmaterial und Verfahren zum Herstellen eines Kompositmaterials und dessen Anwendung als Kondensatorwerkstoff, Anmeldenummer DE 10 2021 206 990.8.

## Figures and Tables

**Table 1:** Measured data of the prepared structured samples. The compositions and equivalent diameters of the BST grains were determined by image analysis from the SEM images. The pellet thickness was measured geometrically, and it was relevant to determine the applied electrical field for a set voltage.

MBO Content (vol%)	Pellet Thickness (mm)	Equivalent Diameter of the BST Grains (µm)
35 ± 2	0.50 ± 0.05	0.50 ± 0.08
55 ± 2	0.55 ± 0.05	0.37 ± 0.07
73 ± 2	0.34 ± 0.05	0.30 ± 0.07

**Table 2:** Measured data of the prepared, unstructured comparison samples. The compositions and equivalent diameters of the BST grains were determined by image analysis from the SEM images. The pellet thicknesses were measured geometrically, and it was relevant to determine the applied electrical field for a set voltage.

MBO Content (vol%).	Pellet Thickness (mm)	Equivalent Diameter of the BST Grains (µm)
40 ± 2	0.70 ± 0.04	0.18 ± 0.04
48 ± 2	0.64 ± 0.05	0.31 ± 0.07
64 ± 2	0.52 ± 0.05	0.25 ± 0.06
73 ± 2	0.51 ± 0.03	0.27 ± 0.06

## Data Availability

All research results relevant to this study are shown in this publication. If there is interest in supplementary information, the authors may be contacted with the given information.

## Appendix A

### Appendix A.1. Calculation

The approximation of tunability in two differently structured capacitor networks, as shown in Figure 2. Only one iteration of tuning was applied, neglecting the redistribution of the tuning voltage due to the tuning itself. The tuning was considered linear with the tuning voltage for the sake of simplicity, and the permittivity of the MBO was neglected. The models relied on the percolation of the BST; otherwise, the MBO could not be neglected anymore.

Assuming the permittivity of the MBO to be 0 and the tunability of the BST to be  1 00⋅rowV,

for the left case (equal distribution) (*C*: capacitance, *U*: voltage, *X_C_*: reactance),

$$C_{total}=\frac{1}{\frac{1}{3C_{part}}+\frac{1}{3C_{part}}+\frac{1}{3C_{part}}}=C_{part}$$

$$U_{any\_row}=\frac{X_{C,\;any\_row}}{X_{C,\;total}}U_{ges}=\frac{1}{3}U_{ges}$$

while for the right case (structured distribution),

$$C_{total}=\frac{1}{\frac{1}{5C_{part}}+\frac{1}{3C_{part}}+\frac{1}{1C_{part}}}=\frac{15}{23}C_{part}$$

$$U_{first\_row}=\frac{X_{C,first}}{X_{C,\;total}}U_{ges}=\frac{\frac{1}{5C_{part}}}{\frac{1}{\frac{15}{23}C_{part}}}U_{ges}=\frac{3}{23}U_{ges}$$

$$U_{second\_row}=\frac{X_{C,second}}{X_{C,\;total}}U_{ges}=\frac{\frac{1}{3C_{part}}}{\frac{1}{\frac{15}{23}C_{part}}}U_{ges}=\frac{5}{23}U_{ges}$$

$$U_{third\_row}=\frac{X_{C,third}}{X_{C,\;total}}U_{ges}=\frac{\frac{1}{1C_{part}}}{\frac{1}{\frac{15}{23}C_{part}}}U_{ges}=\frac{15}{23}U_{ges}$$

From these, the given voltages were calculated. The tunability was (first order) approximated by reducing the capacitance of every row by 1 (% · row)/V · U_row_ and then redoing the above calculation, resulting in the difference of capacitance for the whole block.

The full solution would require infinite iterations of recalculating the tuning of every row from the change in capacitance.

### Appendix A.2. Calculation

Fortran-based user-defined material subroutine (UMAT) to implement the potential gradient dependency of the permittivity. BASE and TUNE are defined material properties. BASE corresponds to the permittivity at zero tuning and TUNE to the tunability at maximum tuning voltage (set to 1 in this model). The temperature gradient DTEMDX(I) at position I reduces the heat transfer (FLUX) at position I down to -(1-TUNE*DTEMDX(I)), therefore simulating a linear tunability.

***SUBROUTINE** UMATHT(U,DUDT,DUDG,FLUX,DFDT,DFDG,*
*1 STATEV,TEMP,DTEMP,DTEMDX,TIME,DTIME,PREDEF,DPRED,*
*2 CMNAME,NTGRD,NSTATV,PROPS,NPROPS,COORDS,PNEWDT,*
*3 NOEL,NPT,LAYER,KSPT,KSTEP,KINC)*
*C*
***INCLUDE** ’ABA_PARAM.INC’*
*C*
***CHARACTER***80 CMNAME*
*DIMENSION DUDG(NTGRD),FLUX(NTGRD),DFDT(NTGRD),*
*1 DFDG(NTGRD,NTGRD),STATEV(NSTATV),DTEMDX(NTGRD),*
*2 TIME(2),PREDEF(1),DPRED(1),PROPS(NPROPS),COORDS(3)*
*C*
*BASE = PROPS(1)*
*TUNE = PROPS(2)*
*C*
***DO** I=1, NTGRD*
*FLUX(I) = (BASE*(TUNE*DTEMDX(I)-1))*DTEMDX(I)*
*DFDG(I,I) = (BASE*(TUNE*DTEMDX(I)-1))*
***END DO***
*C*
***RETURN***
***END***

### Appendix A.3. Calculation

Approximation of the tunability for the layered model. As in Calculation S1, only one tuning iteration was accounted for. Linear tuning and no tuning of the MBO were assumed as well. The permittivity of the MBO could be neglected in this calculation, since the BST did not percolate the structure of interest.

Since the area for each capacitor was equal in the layered model, the variable to be considered was the thickness of each. The ratios of their thicknesses, therefore, were equal to their volume ratios. The respective capacitances resulted in

$$\begin{array}{c} C_{\mathrm{BST}}=\varepsilon_{0}\varepsilon_{r}\left(\mathrm{BST}\right)\frac{A}{\left(1-q\right)d}\\ C_{\mathrm{MBO}}=\varepsilon_{0}\varepsilon_{r}\left(MBO\right)\frac{A}{\left(q\right)d} \end{array}$$

with q being the volume percentage of the MBO and d being the overall thickness of the structure.

From this, the total, non-tuned capacity would be

$$C_{\mathrm{NT},\mathrm{total}}={\left(C_{\mathrm{BST}}^{-1}+C_{\mathrm{MBO}}^{-1}\right)}^{-1}$$

The voltage percentage dropping over the BST sub-capacitance follows as

$$\frac{U_{\mathrm{BST}}}{U_{\mathrm{total}}}=\frac{C_{\mathrm{BST}}^{-1}+C_{\mathrm{MBO}}^{-1}}{C_{\mathrm{BST}}}$$

Assuming a linear dependency of the BST permittivity with the tuning voltage, the effective tuning of the BST results in

$$\tau_{\mathrm{BST},\mathrm{effective}}=\frac{U_{\mathrm{BST}}}{U_{\mathrm{total}}}\tau_{\mathrm{BST},\max}$$

The resulting overall capacitance in the tuned state is therefore

$$C_{T,\mathrm{total}}={\left({\left(C_{\mathrm{BST}}\left(1-\tau_{\mathrm{BST},\mathrm{effective}}\right)\right)}^{-1}+C_{\mathrm{MBO}}^{-1}\right)}^{-1}$$

When plugging in the material capacitances given in the beginning, the dependency shown in Figure 5 follows.

### Appendix A.4. Calculation

Formulas for the material quality factor (MQF) and the commutation quality factor (CQF), with $E_{\max}$ being the maximum applied electrical field strength, τ being the tunability, $\tan\delta_{0}\;$ being the dielectric loss without the applied electrical field, and $\tan\delta_{\max}$ being the dielectric loss at the maximum applied electrical field strength.

$$\mathrm{MQF}\left(E_{\max}\right)=\frac{\tau \left(E_{\max}\right)}{\tan\delta_{0}}$$

$$\mathrm{CQF}\left(E_{\max}\right)=\frac{\tau {\left(E_{\max}\right)}^{2}}{\left(1-\tau \left(E_{\max}\right)\right)\tan\delta_{0}\;\tan\delta_{\max}}$$

**Table A1:** Geometric and dielectric measurement data of the prepared samples. Due to the different thicknesses, the tunabilities were recalculated for the highest electrical field strength, which was applied to all samples (1.57 kV/mm).

MBO Content (%)	Structuring	h (mm)	Dielectric Loss @ 1.1 kV	Tunability @ 1.1 kV (%)	Voltage (V) for 1.57 kV/mm	Tunability @ 1.57 kV/mm (%)	Relative Permittivity @ 0.0 kV/mm (%)
35	struc	0.50	0.013 ± 0.001	50 ± 0.5	785	38.4 ± 0.7	1600 ± 49
40	unstruc	0.70	0.009 ± 0.001	18 ± 2.1	1099	20.9 ± 3.0	1780 ± 37
55	struc	0.55	0.008 ± 0.001	34 ± 0.2	864	26.3 ± 0.3	445 ± 19
48	unstruc	0.64	0.006 ± 0.001	22 ± 0.7	1005	20.4 ± 1.0	547 ± 15
70	struc	0.34	0.006 ± 0.001	38 ± 0.9	534	21.1 ± 1.3	150 ± 17
64	unstruc	0.52	0.004 ± 0.001	19 ± 4.0	816	12.5 ± 6.3	156 ± 15
